# Diagnosis of Cervical Cancer With Parametrial Invasion on Whole-Tumor Dynamic Contrast-Enhanced Magnetic Resonance Imaging Combined With Whole-Lesion Texture Analysis Based on T2- Weighted Images

**DOI:** 10.3389/fbioe.2020.00590

**Published:** 2020-06-11

**Authors:** Xin-xiang Li, Ting-ting Lin, Bin Liu, Wei Wei

**Affiliations:** ^1^Jiangsu Key Laboratory of Molecular and Functional Imaging, Department of Radiology, Zhongda Hospital, Medical School, Southeast University, Nanjing, China; ^2^Department of Radiology, The First Affiliated Hospital of USTC, Division of Life Sciences and Medicine, University of Science and Technology of China, Hefei, China

**Keywords:** cervical cancer, parametrial invasion, DCE-MRI, texture analysis, T2-weighted imaging

## Abstract

**Purpose:** To evaluate the diagnostic value of the combination of whole-tumor dynamic contrast-enhanced magnetic resonance imaging (DCE-MRI) and whole-lesion texture features based on T2–weighted images for cervical cancer with parametrial invasion.

**Materials and Methods:** Sixty-two patients with cervical cancer (27 with parametrial invasion and 35 without invasion) preoperatively underwent routine MRI and DCE-MRI examinations. DCE-MRI parameters (K^trans^, K_ep_, and V_e_) and texture features (mean, skewness, kurtosis, uniformity, energy, and entropy) based on T2-weighted images were acquired by two observers. All parameters of parametrial invasion and non-invasion were analyzed by one-way analysis of variance. The diagnostic efficiency of significant variables was assessed using receiver operating characteristic analysis.

**Results:** The invasion group of cervical cancer demonstrated significantly higher K^trans^ (0.335 ± 0.050 vs. 0.269 ± 0.079; *p* < 0.001), lower energy values (0.503 ± 0.093 vs. 0.602 ± 0.087; *p* < 0.001), and higher entropy values (1.391 ± 0.193 vs. 1.24 ± 0.129; *p* < 0.001) than those in the non-invasion group. Optimal diagnostic performance [area under curve [AUC], 0.925; sensitivity, 0.935; specificity, 0.829] could be obtained by the combination of K^trans^, energy, and entropy values. The AUC values of K^trans^ (0.788), energy (0.761), entropy (0.749), the combination of K^trans^ and energy (0.814), the combination of K^trans^ and entropy (0.727), and the combination of energy and entropy (0.619) were lower than those of the combination of K^trans^, energy, and entropy values.

**Conclusion:** The combination of DCE-MRI and texture analysis is a promising method for diagnosis cervical cancer with parametrial infiltration. Moreover, the combination of K^trans^, energy, and entropy is more valuable than any one alone, especially in improving diagnostic sensitivity.

## Introduction

Cervical cancer is one of the most common malignant diseases of the female reproductive system, and it seriously threatens women's health and life. Accurate preoperative staging of cervical cancer plays an important role in clinical treatment decisions and prognosis. As a matter of principle, surgery is performed for cervical cancer without parametrial involvement, while tumors with parametrial invasion are treated with radio-chemotherapy. To the best of our knowledge, cervical cancer with parametrial invasion is closely related to recurrence and survival after treatment (Chung et al., 2010; Munagala et al., 2010; Noh et al., 2014; Kong et al., 2016; Xia et al., 2016; Dai et al., 2018). Therefore, accurate diagnosis of cervical cancer with parametrial invasion is of great clinical significance. Parametrial invasion is usually evaluated by conventional magnetic resonance (MR) imaging and gynecological examination. Several previous investigations showed that traditional imaging features, such as full-thickness disruption of the normal cervix stroma with nodular or spiculated lesions extending to the adjacent parametrium on T2-weighted images, were considered to be parametrial invasion (Freeman et al., 2012; Patel-Lippmann et al., 2017); however, image analysis is a subjective procedure with low interobserver agreement. An objective and quantitative method for evaluating parametrial infiltration in clinical practice is needed.

Currently, many new techniques have been applied at the molecular level, such as deep learning, proteomics, and protein interaction network (Wang et al., 2019, 2020; Deng et al., 2020; Hu et al., 2020). Besides, several imaging techniques (Park et al., 2014; Zhou et al., 2016) and radiomics (Mu et al., 2015; Meng et al., 2017) have been reported in the assessment of patients with parametrial invasion to determine the stage and treatment of cervical cancer. Chiappa et al. reported that 3D ultrasound volumes can be used to more precisely define the location and degree of cervical cancer invasion (Chiappa et al., 2015). Several studies show that the apparent diffusion coefficient (ADC) value of cervical cancer is significantly lower in cancer with parametrial invasion than in cancer without parametrial invasion (Park et al., 2014; Woo et al., 2018). Park et al. reported that merging high b-value diffusion-weighted MR imaging with background body signal suppression and T2-weighted high-spatial-resolution imaging could improve diagnostic efficiency of predicting cervical cancer with parametrial infiltration (Park et al., 2014). Recently, histogram analysis of ADC has shown potential to predict outcomes after concurrent chemo-radiotherapy in patients with cervical cancer (Meng et al., 2017). Moreover, the study showed that tracer uptake heterogeneity in tumors characterized by texture features based on fluorodeoxyglucose-positron emission tomography (18-FDG PET) is highly relevant to the stage of cervical cancer (Mu et al., 2015). To our knowledge, however, no reported studies demonstrated the role of dynamic contrast-enhanced magnetic resonance imaging (DCE-MRI) or texture analysis in evaluating cervical cancer with parametrial invasion.

The purpose of our study was to investigate the diagnostic value of the combination of whole-tumor volumetric DCE-MRI and texture features based on T2-weighted images for predicting parametrial invasion. These quantitative parameters might improve the diagnostic accuracy of cervical cancer with parametrial infiltration prior to treatment.

## Materials and Methods

### Patients

This study was approved by our institutional review board and the patients provided written informed consent to participate. Seventy-five patients with histopathologically confirmed cervical cancer were admitted to our hospital from September 2017 to April 2019. Routine MRI sequences and DCE-MRI examinations were preoperatively performed. Thirteen patients were excluded according to the following criteria: (1) tumor diameter was <1 cm (*n* = 7); (2) image quality was not available for the next analysis (*n* = 4); (3) diameter of the necrotic lesions in the tumor was more than 5 mm (*n* = 2). Finally, 62 patients (25–56 years old, mean age 45 years) were eligible for the study (27 with parametrial invasion and 35 without invasion), including FIGO stage IA (*n* = 14), IB (*n* = 9), IIA (*n* = 12), IIB (*n* = 15), IIIA (*n* = 7), and IIIB (*n* = 5).

### Imaging Acquisition

MRI was acquired by using a 3.0 T Signa HDxT MRI machine (GE Healthcare, USA) with an 8-channel phased array body coil. The scanning program for the pelvis was as follows: unenhanced axial T1-weighted imaging, axial and sagittal T2-weighted imaging, axial T2-weighted imaging with fat saturation, axial diffusion weighted imaging, axial DCE-MRI, and axial and sagittal contrast-enhanced T1-weighted imaging with fat saturation.

T2-weighted imaging was obtained using a fast spin echo sequence. The following protocol was used: repetition time (TR)/echo time (TE), 4600/30 ms; number of excitations, 2; section thickness, 6 mm; intersection gap, 2 mm; field of view (FOV), 240 × 240 mm; matrix size, 320 × 256; and total time, 2 min and 14 s.

DCE-MRI was obtained using T1-weighted fat-suppression images and a three dimensional (3D) liver acceleration volume acquisition (LAVA) sequence during the injection of 0.1 mmol/kg of gadodiamide (Omniscan, GE Healthcare, USA) at a rate of 2 ml/s and following a 20-mL saline flush at the same rate. The contrast medium was injected after the acquisition of three sets of pre-contrast T1 mapping using three flip angles: 5, 10, and 15 (total: 43 dynamics). The following protocol was used: TR/TE, 4.2/2.2 ms; 15° flip angle; FOV, 380 × 340 mm; matrix size, 320 × 224; section thickness, 4 mm; time resolution, 7.0 s; and total time, 4 min, 40 s.

### Imaging Analysis and Parameter Acquisition

DCE-MRI data were processed by Omni-Kinetics (O.K.; GE Healthcare, China) software. Multi-flip angle T1 mapping transformed the signal intensity into contrast agent concentration. For the evaluation of the arterial input function (AIF), a region of interest (ROI) was manually placed on the iliac artery. Tumors were outlined on each slice from DCE-MRI images in order to show the volume of interest (VOI) of the whole tumor. The DCE-MRI parameters (K^trans^ [the volume transfer constant], K_ep_ [the flux rate constant], and V_e_ [fractional extravascular extracellular space volume]), were calculated using a modified Tofts model.

Texture features were acquired by axial T2-weighted images with ITK-SNAP software (version 3.6.0) and O.K. software. For each patient, the VOI was calculated using ITK-SNAP software by manually delineating the ROI along the edge of the tumor on each slice for the entire tumor by referencing the corresponding contrast-enhanced images. Texture features were extracted from the delineated VOI by the O.K. software.

DCE-MRI and texture features were independently measured by two radiologists using the O.K. software (XX.L. and TT.L. with 6 years and 12 years of clinical experience, respectively, in gynecologic oncology MR imaging). Reader 1 measured DCE-MRI and texture features twice in 1 week to estimate intraobserver reproducibility, and his first measurement was compared with the measurement obtained by reader 2 to assess interobserver agreement. The mean of the two measured data of reader 1 was statistically analyzed. The intraclass correlation coefficient (ICC) of more than 0.75 edindicated good agreement.

### Statistical Analysis

Quantitative metrics were indicated as mean ± standard error, and the normality test was assessed using the Kolmogorov-Smirnov method. The normal distribution of the DCE-MRI and texture feature parameters data was compared between the invasion group and the non-invasion group was using one-way analysis of variance; comparisons among the metrics of each group were processed using least significant difference (LSD) test. Non-normal distribution of data were compared using the Mann–Whitney test. Receiver operating characteristic (ROC) curve analysis was used to assess the diagnostic ability of DCE-MRI and texture feature parameters in diagnosing parametrial invasion. The AUC was compared using the Delong Clarke-Pearson method (DeLong et al., 1988). Cut-off values were obtained by maximizing Youden's index (sensitivity+specificity-1). Statistical analysis was performed using SPSS 23.0 (IBM Corp., Armonk, NY) and GraphPad Prism 8.0 (GraphPad software, San Diego, CA). P < 0.05 was considered as the threshold for statistical significance.

## Results

Excellent intra- and interobserver agreements were found in the measurements of DCE-MRI and texture features metrics (Table 1). The intra- and interobserver ICCs of K^trans^, K_ep_, and V_e_ were 0.917 and 0.841, 0.934 and 0.893, and 0.842 and 0.927, respectively. Meanwhile, the intra- and interobserver ICCs of the mean value were 0.924 and 0.858, of skewness were 0.925 and 0.915, of kurtosis were 0.826 and 0.935, of uniformity were 0.904 and 0.912, of energy were 0.879 and 0.892, and of entropy were 0.924 and 0.921.

**Table 1:** Comparison of DCE-MRI and texture feature derived parameters.

**Parameters**	**Invasion group (*n* = 27)**	**Non-invasion group (*n* = 35)**	***P*-value**	**ICC**	
				**Inter (95% CI)**	**Intra (95% CI)**
**DCE-MRI**					
K^trans^, min^−1^	0.335 ± 0.050	0.269 ± 0.079	<0.001	0.917 (0.834, 0.953)	0.841 (0.792, 0.878)
K_ep_, min^−1^	0.538 ± 0.103	0.526 ± 0.110	0.652	0.934 (0.869, 0.958)	0.893 (0.847, 0.961)
V_e_	0.538 ± 0.095	0.511 ± 0.104	0.270	0.842 (0.793, 0.902)	0.927 (0.842, 0.972)
**Texture**					
Mean	679.53 ± 66.02	697.12 ± 59.70	0.260	0.924 (0.835, 0.959)	0.858 (0.786, 0.919)
Skewness	0.043 ± 0.242	0.085 ± 0.267	0.502	0.925 (0.834, 0.946)	0.915 (0.862, 0.968)
Kurtosis	3.252 ± 0.477	3.263 ± 0.653	0.940	0.826 (0.783, 0.879)	0.935 (0.883, 0.969)
Uniformity	0.912 ± 0.013	0.915 ± 0.034	0.719	0.904 (0.837, 0.961)	0.912 (0.852, 0.969)
Energy	0.503 ± 0.093	0.602 ± 0.087	<0.001	0.879 (0.802, 0.948)	0.892 (0.817, 0.948)
Entropy	1.391 ± 0.193	1.24 ± 0.129	<0.001	0.924 (0.846, 0.958)	0.921 (0.836, 0.971)

*DCE-MRI, dynamic contrast-enhanced magnetic resonance imaging; K^trans^, volume transfer constant; K_ep_, flux rate constant between extravascular extracellular space and blood plasma; V_e_, fraction of extravascular extracellular volume*.

**Figure 1:**
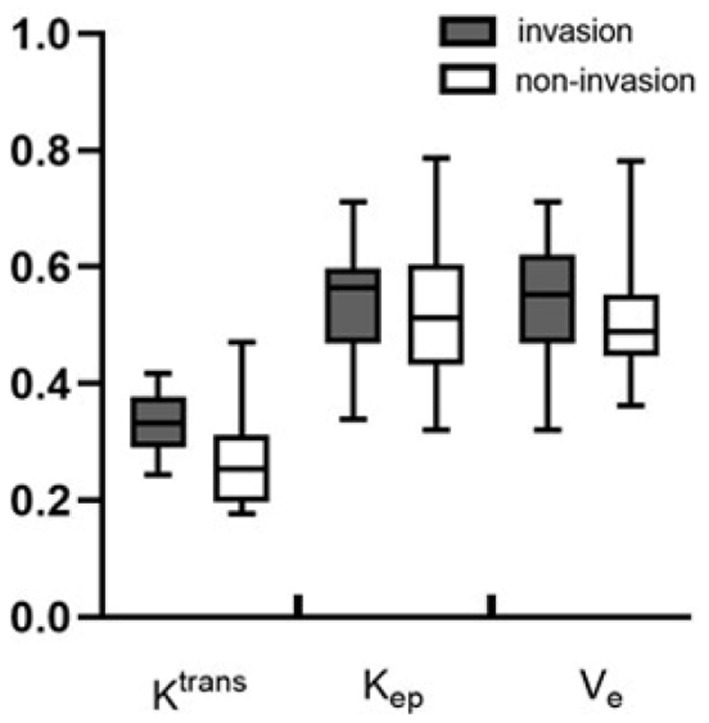
Boxplots of the DCE-MRI parameters of K^trans^, K_ep_, and V_e_ between the invasion and non-invasion groups.

**Figure 2:**
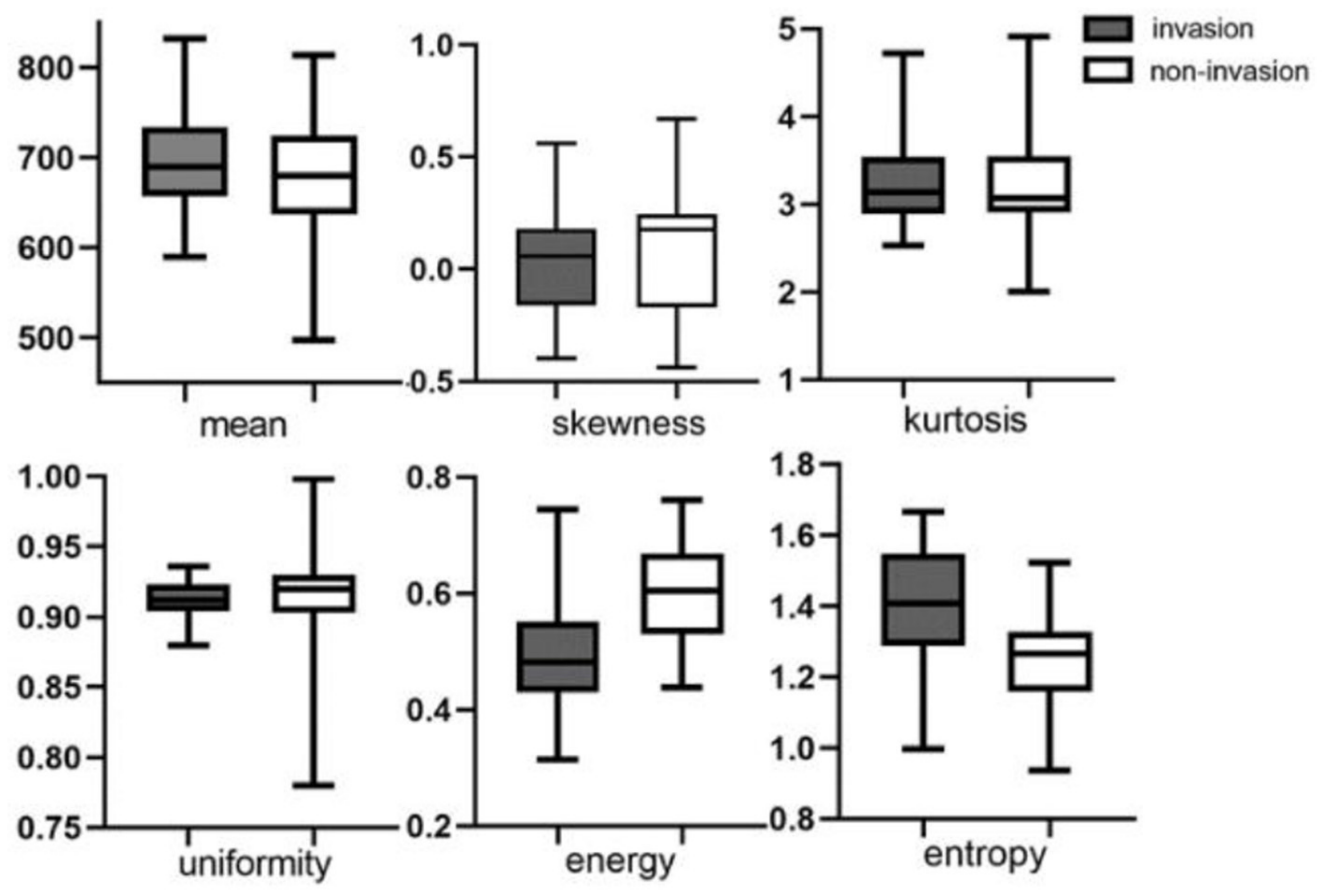
Boxplots of texture features of mean value, skewness, kurtosis, uniformity, energy, and entropy between invasion and non-invasion groups.

Metrics derived from DCE-MRI and texture features were compared between the invasion group and the non-invasion group and are summarized in Table 1 and Figures 1, 2. The invasion group indicated a significantly higher K^trans^ (0.335 ± 0.050 vs. 0.269 ± 0.079; *p* < 0.001), lower energy values (0.503 ± 0.093 vs. 0.602 ± 0.087; *p* < 0.001), and higher entropy values (1.391 ± 0.193 vs. 1.24 ± 0.129; *p* < 0.001) than the non-invasion group, while there was no significant difference for K_ep_, V_e_, mean, skewness, kurtosis, or uniformity.

**Figure 3:**
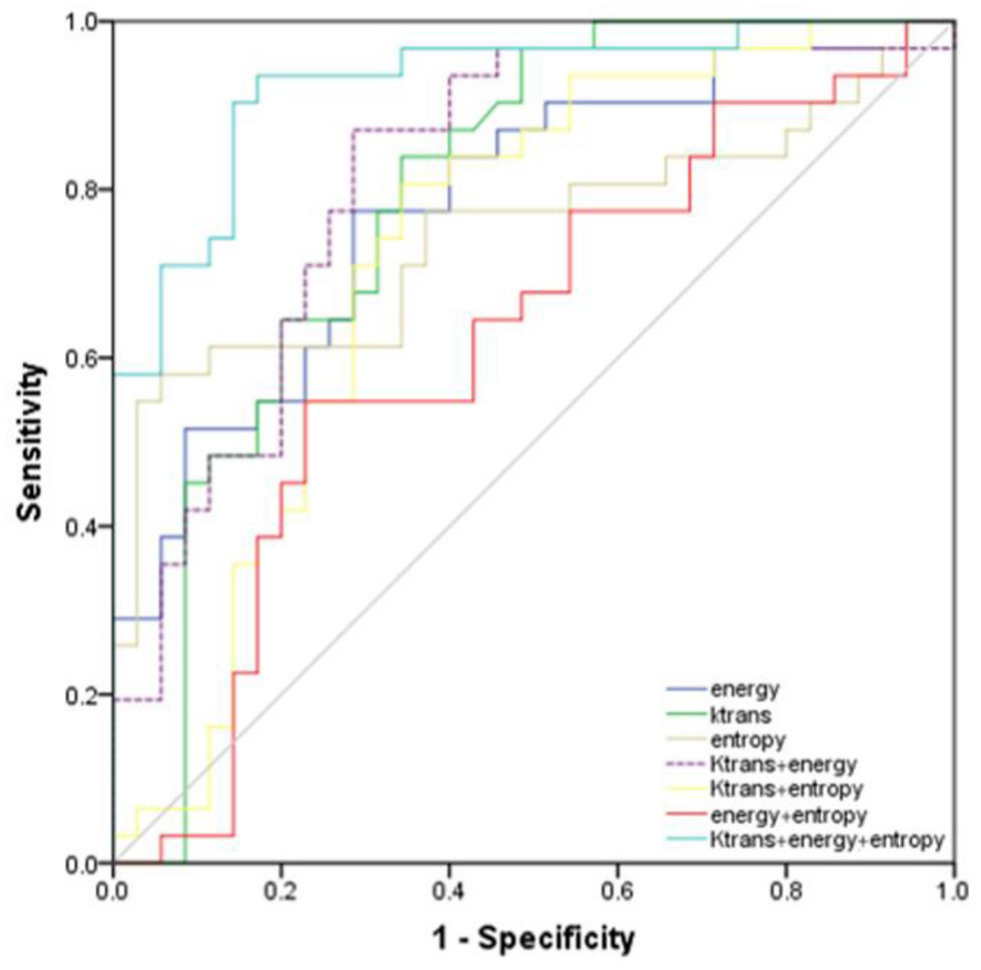
Receiver operating characteristic curves of the energy, K^trans^, entropy, combination of K^trans^ and energy, combination of K^trans^ and entropy, combination of energy and entropy, combination of K^trans^, energy, and entropy for diagnosis of invasion and non-invasion of cervical cancer.

**Figure 4:**
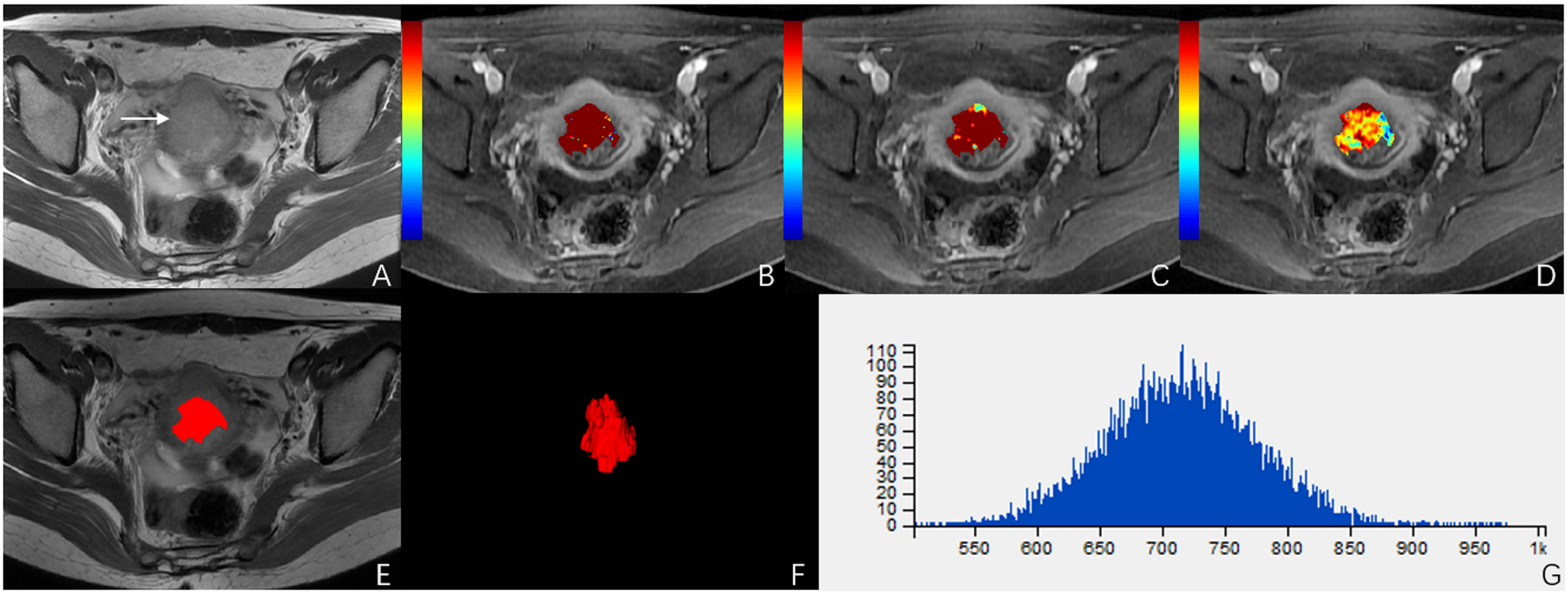
A 51-year-old woman with stage IIB cervical cancer. **(A)** T2-weighted image (T2WI) shows a slightly hyperintense cervical mass. **(B–D)** K^trans^, K_ep_, and V_e_ parametric maps are derived from DCE-MRI. The corresponding values are 0.577/min, 0.639/min, 0.694. **(E,F)** The volume of the tumor is drawn from T2WI. **(G)** Histogram map of the entire tumor.

**Figure 5:**
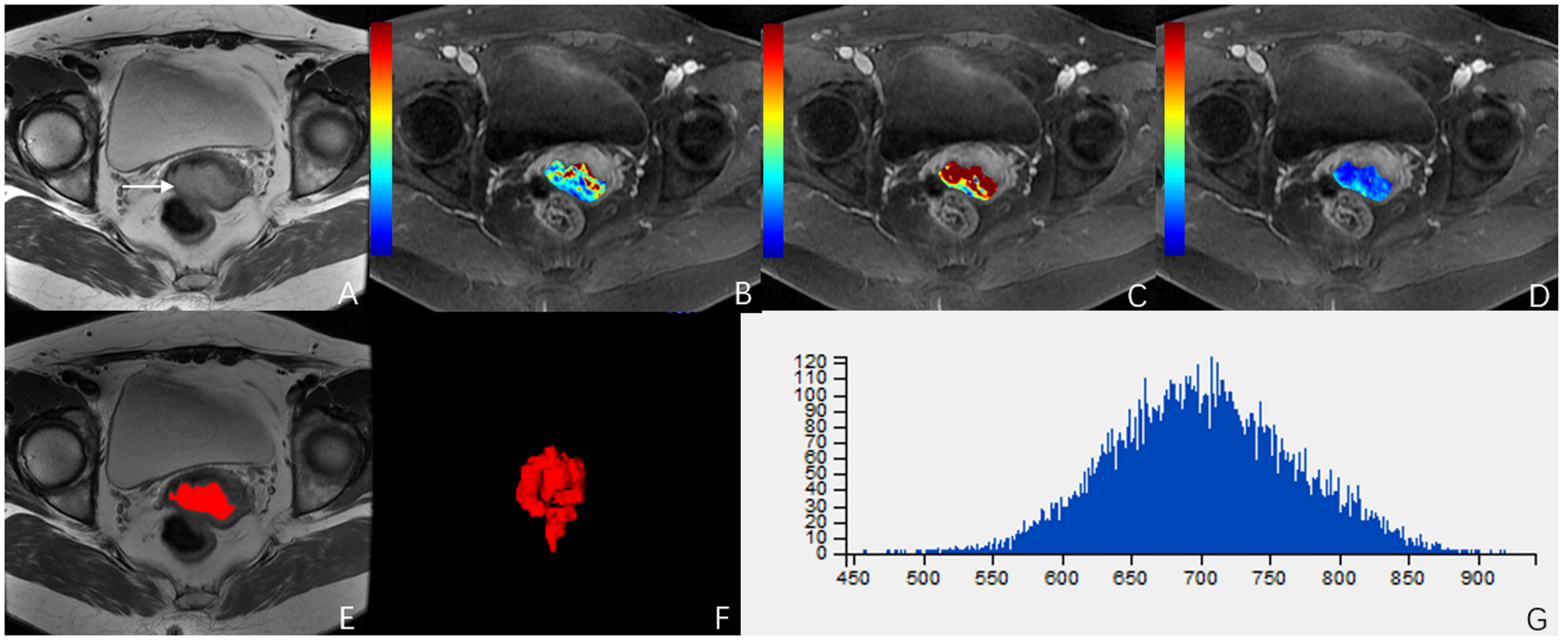
A 49-year-old woman with stage IIA cervical cancer. **(A)** T2-weighted image (T2WI) shows a slightly hyperintense cervical mass. **(B–D)** K^trans^, K_ep_, and V_e_ parametric maps are derived from DCE-MRI. The corresponding values are 0.196/min, 0.430/min, 0.396. **(E,F)** The volume of the tumor is drawn from T2WI. **(G)** Histogram map of the entire tumor.

ROC analysis showed that setting the K^trans^ cut-off value to ≥0.286 min^−1^ produced the best diagnostic performance for diagnosing parametrial infiltration (AUC, 0.788; sensitivity, 0.839; specificity, 0,657). The best diagnostic ability could be obtained by setting the threshold value of energy at ≤0.488 (AUC, 0.761; sensitivity, 0.710; specificity, 0.714). Setting the critical value of entropy at ≥1.387 obtained the best diagnostic index (AUC, 0.749; sensitivity, 0.581; specificity, 0.943). The combination of K^trans^ and energy had a significantly better diagnostic index than an independent diagnosis of K^trans^, energy, and entropy (*p* = 0.036, *p* = 0.029, *p* = 0.047). However, using a combination of K^trans^ and entropy (AUC, 0.727; sensitivity, 0.806; specificity, 0.657) and a combination of energy and entropy (AUC, 0.619; sensitivity, 0.548; specificity, 0.771) as the diagnostic marker achieved a significantly worse performance than other single and combination parameters. The combination of K^trans^, energy, and entropy (AUC, 0.925; sensitivity, 0.935; specificity, 0.829) resulted in a significantly better diagnostic performance than K^trans^, energy, entropy, a combination of K^trans^ and energy, a combination of K^trans^ and entropy, or a combination of K^trans^ and energy (*p* = 0.042, *p* = 0.037, *p* = 0.018, *p* = 0.048, *p* = 0.007, *p* = 0.029, respectively) (Figure 3). Table 2 shows the detailed diagnostic performances. The representative images of DCE-MRI and texture features of cervical cancer with and without invasion are summarized in Figures 4, 5.

**Table 2:** Diagnostic efficiency of each parameter and their combined metrics.

**Parameters**	**Cut-off value**	**AUC**	**Sensitivity**	**Specificity**
K^trans^, min^−1^	0.286	0.788 (0.690–0.849)	0.839	0.657
Energy	0.488	0.785 (0.717–0.857)	0.774	0.714
Entropy	1.387	0.749 (0.657–0.828)	0.581	0.943
K^trans^ + energy		0.813 (0.748–0.859)	0.871	0.714
K^trans^ + entropy		0.728 (0.604–0.830)	0.806	0.657
Energy + entropy		0.619 (0.481–0.757)	0.548	0.771
K^trans^ + energy + entropy		0.925 (0.853–0.976)	0.935	0.829

*Data in parentheses indicate 95% confidence intervals. AUC, area under curve; K^trans^, volume transfer constant*.

## Discussion

The signal intensity kinetics acquired by DCE-MRI suggest the underlying microvessel density, perfusion, permeability, and the extracellular-extravascular space composition of tumors (Zahra et al., 2007; Bonekamp et al., 2016). DCE-MRI can predict the response to and outcomes of radiotherapy in patients with cervical cancer (Tao et al., 2019). Tao et al. reported (2019) that the K^trans^ of high-grade ductal carcinoma *in situ* of the breast is higher than that of low-grade ductal carcinoma. Li et al. (2015) reported that the K^trans^ of high-grade glioma is higher than that of low-grade glioma. stoLikewise, the present study infound that the K^trans^ value of the invasive group was higher than that of the non-invasive group. The parameter K^trans^ reflected tumor angiogenesis, which is proportional to the density of the tumor vessels. This indicated that the angiogenesis of the invasive cervical cancer group was greater than that of the non-invasive group, and the malignant degree of the invasive group was higher than that of the non-invasive group. The more malignant the tumors are, the more angiogenesis they have. Early cervical cancer consists mainly of neovascularization, but the blood vessels are few in number and have low permeability. The growth rate of advanced cervical cancer is faster than that of early cervical cancer, and the tumor's demand for blood oxygen is increased, so that a large number of new blood vessels are formed. The angiogenesis and the permeability of the blood vessels is increased. The tortuous course of the blood vessels increases their permeable area. Moreover, the endothelial cells of blood vessels are irregular. Therefore, the contrast agent permeates through the gaps in the blood vessels more easily than it does in early cervical cancer. We can conclude that the parameters of K^trans^ in the infiltration group were higher than those in the non-infiltration group.

Texture analysis is a new image post-processing computer technology that quantitatively analyzes the distribution rules and characteristics of image pixels and reflects lesion heterogeneity and the fine differences of tumors. Energy reflects uniformity and texture the coarseness of the images. The better distributed the gray of the image is, the greater its value. In this study, the energy value of the non-invasive group was larger than that of the invasive group, which indicated that the images of the invasive group were less uniform than those of the non-invasive group. This may be owing to the cystoid degeneration and necrosis in the invasive cervical cancer group. Entropy, reflecting the basic degree of chaos in the gray levels, is a measure of the image information. It is mainly used to evaluate the uniformity of image texture. The entropy value of the parametrial infiltration group was higher than that of the non-infiltration group, which indicated that the distribution of image pixels in the infiltration group was more discrete and disordered than that in the non-infiltration group. The reason for this difference may be related to the degree of malignancy. Several studies showed that tumors with a high degree of malignancy have high heterogeneity (Ng et al., 2013; Zhang et al., 2017a,b), and a high entropy value represents high tumor heterogeneity (Guan et al., 2017), and this is in accordance with our study showing that advanced cervical cancer representing high heterogeneity has high entropy. Guan et al. (2017) showed that cervical cancers with higher (IIB-IVA) rather than lower (IB-IIA) FIGO stages had lower energy and higher entropy of texture features based on ADC images. This result is consistent with our study showing that advanced cervical cancer has lower energy and higher entropy than early stage cervical cancer. The mean value, skewness, kurtosis, and homogeneity had no statistical significance in diagnosing cervical cancer with parametrial infiltration. These parameters may have significance if we performed multiple sequences of MRI for texture analysis.

In the present study, K^trans^, energy, entropy, and combinations of them had the optimal diagnostic performance for diagnosing cervical cancer with parametrial infiltration; particularly, the combination of K^trans^, energy and entropy had the highest AUC (0.925) and sensitivity (93.5%). This indicated that the combination of K^trans^, energy, and entropy was more significant than the other parameters for the diagnosis of parametrial infiltration. In other words, DCE-MRI, representing quantitative perfusion information at the molecular level, and texture features, representing a mathematical model of the gray distribution of quantitative image pixels, are the most valuable for the diagnosis of cervical cancer with parametrial infiltration. Thus, we can use more accurate quantitative parameters at the microscopic level instead of making a subjective diagnosis with a larger margin of error to evaluate parametrial infiltration. Quantitative parameters can be used as an important ancillary diagnostic tool for routine MR examination and can provide a reference for the establishment of an artificial intelligence prediction model of cervical cancer with parametrial infiltration.

Several limitations of the present study are as follows. First, the sample size of this study was relatively small. Second, the ROIs of the tumors were manually performed, which might increase the variability of the data measurement. Third, our study did not distinguish the pathological types of cervical cancer, such as squamous cell carcinoma, adenocarcinoma, and small cell carcinoma.

In conclusion, this study showed that the invasion group of cervical cancer demonstrated significantly higher K^trans^, lower energy values, and higher entropy values than those in the non-invasion group. Both DCE-MRI and texture analysis were valuable in the diagnosis. A combination of DCE-MRI and texture analysis may be a promising method to improve accuracy in diagnosing cervical cancer with parametrial infiltration prior to treatment and has great significance in the medical field.

## Data Availability Statement

All datasets generated for this study are included in the article/supplementary material.

## Ethics Statement

The studies involving human participants were reviewed and approved by Medical research ethics board of the First Affiliated Hospital of USTC, Division of Life Sciences and Medicine, University of Science and Technology of China. The patients/participants provided their written informed consent to participate in this study. Written informed consent was obtained from the individual(s) for the publication of any potentially identifiable images or data included in this article.

## Author Contributions

WW, TL, and BL contributed conception and design of the study. XL organized the database, performed the statistical analysis, and wrote the first draft of the manuscript. WW, XL, TL, and BL wrote sections of the manuscript. All authors contributed to manuscript revision, read and approve the submitted version.

## Conflict of Interest

The authors declare that the research was conducted in the absence of any commercial or financial relationships that could be construed as a potential conflict of interest.
